# Factors associated with deferral or non‐performance of an organized breast cancer screening program during the COVID‐19 pandemic in France

**DOI:** 10.1002/cam4.7444

**Published:** 2024-08-27

**Authors:** Jean Papadacci Stephanopoli, Leila Bouazzi, Myriam Guerbaz‐Sommi, Olivier Graesslin, Aline Hurtaud, Salvatore Ilardo, Jan Chrusciel, Cécile Barbaret, Camille Bertrand, Stéphane Sanchez

**Affiliations:** ^1^ Department of General Medicine Université de Reims Champagne‐Ardenne Reims Marne France; ^2^ Médecine générale Maison de Santé Pluriprofessionnelle de la Forêt d'Orient Piney Aube France; ^3^ Comité Universitaire de Ressources pour la Recherche en Santé, Université de Reims Champagne‐Ardenne Reims Marne France; ^4^ CRCDC Centre Régional de Coordination des Dépistages des Cancers du Grand Est Troyes Aube France; ^5^ Department of Gynecology and Obstetrics, Centre Hospitalier Universitaire de Reims Université de Reims Champagne‐Ardenne Reims Marne France; ^6^ Department of Public Health and Performance Centre Hospitalier de Troyes Troyes France; ^7^ Univ. Grenoble Alpes, CNRS, CHU Grenoble Alpes, Grenoble Institute of Engineering, TIMC‐IMAG, Univ. Grenoble Alpes, Grenoble isère France

**Keywords:** organized programs, screeningbreast cancer, COVID‐19, healthcare promotion, screening

## Abstract

**Background:**

Delays in detection and treatment of breast cancer can lead to increased mortality. To date, participation in organized breast cancer screenings (OBCS) has been suboptimal worldwide. The objective of this study was to investigate the factors associated with deferral or non‐performance of mammography during the COVID‐19 pandemic for women who had previously participated in OBCS.

**Methods:**

A retrospective observational study was conducted on a cohort of 6282 women from the Aube Department of France, who were invited to an OBCS in 2020. Participants were divided into women who promptly underwent screening after receiving an invitation (between 22 and 25 months elapsed since the last mammogram), women who underwent late screening (≥26 months since the last mammogram), and those who were never screened. Data were collected from a self‐reported questionnaire. Comparative and multivariable analyses modeling the probability of each type of attendance were performed using these data.

**Results:**

In total, 2301 women (aged 50–74 years) returned a valid questionnaire. Compared to women who promptly underwent mammography, non‐ and late‐screening participants were younger, had less frequent gynecological follow‐up and a less frequent history of colorectal cancer screening. Women with higher education status and those residing in socially disadvantaged areas were more likely to attend late.

**Conclusion:**

The absence of regular gynecological follow‐up and the absence of colorectal cancer screening were significant factors associated with deferral of or non‐attendance at OBCS.

## 1 INTRODUCTION

Breast cancer is the most common cancer among women worldwide.^1^ In 2020, it accounted for an estimated 2.3 million new cases and was the leading cause of cancer‐related deaths in women.^1^ Breast cancer is particularly frequent in high‐income countries,^2^ although mortality associated with this cancer is declining in these countries.^3^ The improved survival and declining mortality are largely due to the implementation of organized breast cancer screening (OBCS) which enables detection of breast cancer at an early stage.^1^, ^3^, ^4^ OBCS programs may differ according to national or regional guidelines.^5^, ^6^, ^7^, ^8^

Three types of organized cancer screenings currently coexist in France, namely breast cancer, colorectal cancer, and cervical cancer screening.^9^, ^10^ The breast cancer screening program is the oldest (it was first introduced in 1994 and was extended to the whole country in 2004). OBCS consists in undergoing a bilateral mammography once every 2 years from the age of 50 to 74 years.^11^, ^12^ The first invitation was sent by the local antenna of the National Health Insurance organization to women aged 50, by postal mail or via the woman's online health account. A first reminder was sent after 6 months, and a second reminder after another 6 months. It has been clearly established that OBCS reduces mortality in countries where such programs have been implemented.^13^, ^14^, ^15^, ^16^ However, the rate of participation in OBCS programs remains suboptimal. In France, the maximum rate of participation was 52.4% in 2011 to 2012. Participation had declined to 48.5% in 2019. The participation rate fell to 42.6% in 2020 (at the start of the COVID‐19 pandemic), before rising again to reach 50.6% in 2021.^17^

The Aube Department is a rural department with a population of 311,435 according to the 2020 census.^18^ The OBCS participation rate of the department was 54.8% in 2021,^19^ which was higher than the nationwide participation rate, but lower than the 70% to 75% target mentioned by European guidelines for quality assurance in breast cancer screening and diagnosis.^20^

While some northern European countries have reached participation levels above the target (>80% in Finland), numerous other European countries have participation levels well below the target.^21^ Some studies have shown that a delay of 1 to 3 months in breast cancer detection can be enough to increase breast cancer‐related, and all‐cause mortality.^22^, ^23^, ^24^

The recent COVID‐19 pandemic has had a profound impact on the global delivery of OBCS services. A meta‐analysis showed that screening and diagnosis of breast cancer decreased by 41% to 53% and 18% to 29% between 2019 and 2020 respectively, with the most marked declines observed in countries that implemented lockdowns during the pandemic.^25^ In this context, an understanding of the factors associated with declining rates of OBCS participation rates could help to better target efforts to boost participation. The primary objective of this study was to describe the factors associated with late or non‐participation in OBCSs during the COVID‐19 pandemic in the Aube department of France, among women who had previously participated. Secondary objectives were to investigate whether these participants considered their deferral or non‐performance were related to the COVID‐19 pandemic.

## 2 METHODS

### 2.1 Study design

A retrospective observational study based on a self‐reported questionnaire was conducted in 2022, among a population of women living in the Aube Department in France and who received an invitation to undergo a mammogram in 2020 from the regional center that was in charge of coordinating the OBCS.^26^ The invitation period for participants was from 1 June 2020 to 31 December 2020 (after the first French lockdown implemented for the COVID‐19 pandemic). The self‐report questionnaire was sent to all included participants in June 2022 with an informational leaflet and a postage‐paid return envelope. Participants were asked to return the filled‐out questionnaire within 2 months. A reminder was sent to women who had not responded after 1 month.

### 2.2 Study population

Participants were selected from a database compiled by the regional center for the coordination of the OBCS. Inclusion criteria were: (i) women who were invited to participate in the OBCS between 1 June and 31 December 2020, (ii) who had participated in prior OBCS programs (particularly in the second semester of 2018 or in 2019), and (iii) whose prior mammogram findings were classed as ACR1 (normal) or ACR2 (benign abnormalities)^27^ allowing them to remain eligible for continued screening in 2020.

Exclusion criteria were: (i) the absence of any indication for OBCS (patients at high or very high risk requiring specific, individual screening procedures), (ii) women who had never previously received an invitation and/or participated in an OBCS program, (iii) women whose invitations were delayed during the initial lockdown period (March, April and May 2020), (iv) women under any form of legal protection, v) women who no longer lived at the recorded residential address, and (vi) those who had died. After having sent out the questionnaire, a second round of exclusions was added and excluded those who: (i) could not be re‐contacted at the given residential address (returned mail), (ii) did not consent to the use of their data for research purposes, (iii) had not returned the filled questionnaire within 2 months of receiving it despite the reminder, and (iv) whose returned questionnaires were incomplete.

### 2.3 Study endpoints

The primary endpoint of the study was to measure prompt, late or non‐attendance for the OBCS‐related mammography. Prompt attendance was defined as having had a mammography between 22 and 25 months after the previous one. Late attendance was defined as ≥26 months elapsed since the date of the last OBCS mammography, based on data in the literature^22^, ^23^, ^24^ and considering a waiting time of 4 months to obtain an appointment, which can be considered long in the context of the French Healthcare system (an invitation that was sent out at 22 months after the date of the previous OBCS appointment). All women who did not attend the mammography after the OBCS invitation was sent out were classified as participants with no attendance. The study end date for non‐attendance was May 2022.

### 2.4 Data collection

Data were obtained from three sources: the database of the regional center for the coordination of the OBCS in the greater eastern region of France, the national statistics database, and the self‐reported questionnaires. All data were anonymized for analysis. Data extracted from the first database included the age of the participants at the time of invitation in 2020; the date of their previous OBCS mammography in the 2018 to 2019 round of screening; the date of the mammogram performed subsequent to the 2020 invitation (if performed); whether or not the woman had ever previously participated in organized screening for colorectal cancer; and the postal code of residence. Data obtained from the national statistics database included the social deprivation index,^28^ a score indicating the accessibility to healthcare services on a local level,^29^ and the population density of the place of residence.

### 2.5 Self‐report questionnaire

The self‐report questionnaire comprised 15 questions, of which 8 called for binary answers and 7 were multiple‐choice questions (Table S1). Questions included the participants' educational, socio‐professional, and marital status at the time they received the OBCS invitation, whether they had children or not, as well as their nationality. Responses regarding medical follow‐up data were recorded, including the details of their declared general practitioner (GP), whether their GP recommended participation in OBCS, having their own motorized transportation, the length of time required to travel to their nearest mammography provider, any history of cancer other than breast cancer and whether any close relatives and friends have or have had breast cancer. The last question (“Please indicate the reason(s) why you have not yet attended, or why you attended the mammography late”) was solely directed to the women who had performed their mammography late or not at all, and several answers were possible.

### 2.6 Statistical analysis

Categorical variables were expressed as number and percentages (%) and compared using the Chi‐squared test or Fisher's exact test, as appropriate. Continuous variables were expressed as mean ± standard deviation (SD), or median and interquartile range (first quartile Q1 and third quartile Q3) and compared using the analysis of variance (ANOVA). Non‐collinear variables yielding a *p*‐value < 0.20 by univariate analysis were included in a multinomial multivariable logistic regression model, using participants who promptly underwent OBCS as the reference group. Variables included in the model were further selected by expert review based on clinical relevance. Results were expressed as Odds Ratios (OR) with 95% confidence intervals (CI). All analyses were performed using SAS version 9.4 (SAS Institute Inc., Cary, NC, USA) and *p*‐values < 0.05 were considered statistically significant.

### 2.7 Ethical considerations

This study was performed in accordance with the Declaration of Helsinki. Since this study was a retrospective, non‐interventional, observational study, it falls outside the scope of studies requiring Ethics Committee approval according to French legislation.^30^ The study was declared to the French national data privacy commission, *Commission Nationale de l'Informatique et des Libertés* (CNIL) and was approved by the Scientific Commission of the regional center for the coordination of OBCS for the greater eastern region of France in February 2022.

## 3 RESULTS

**FIGURE 1:**
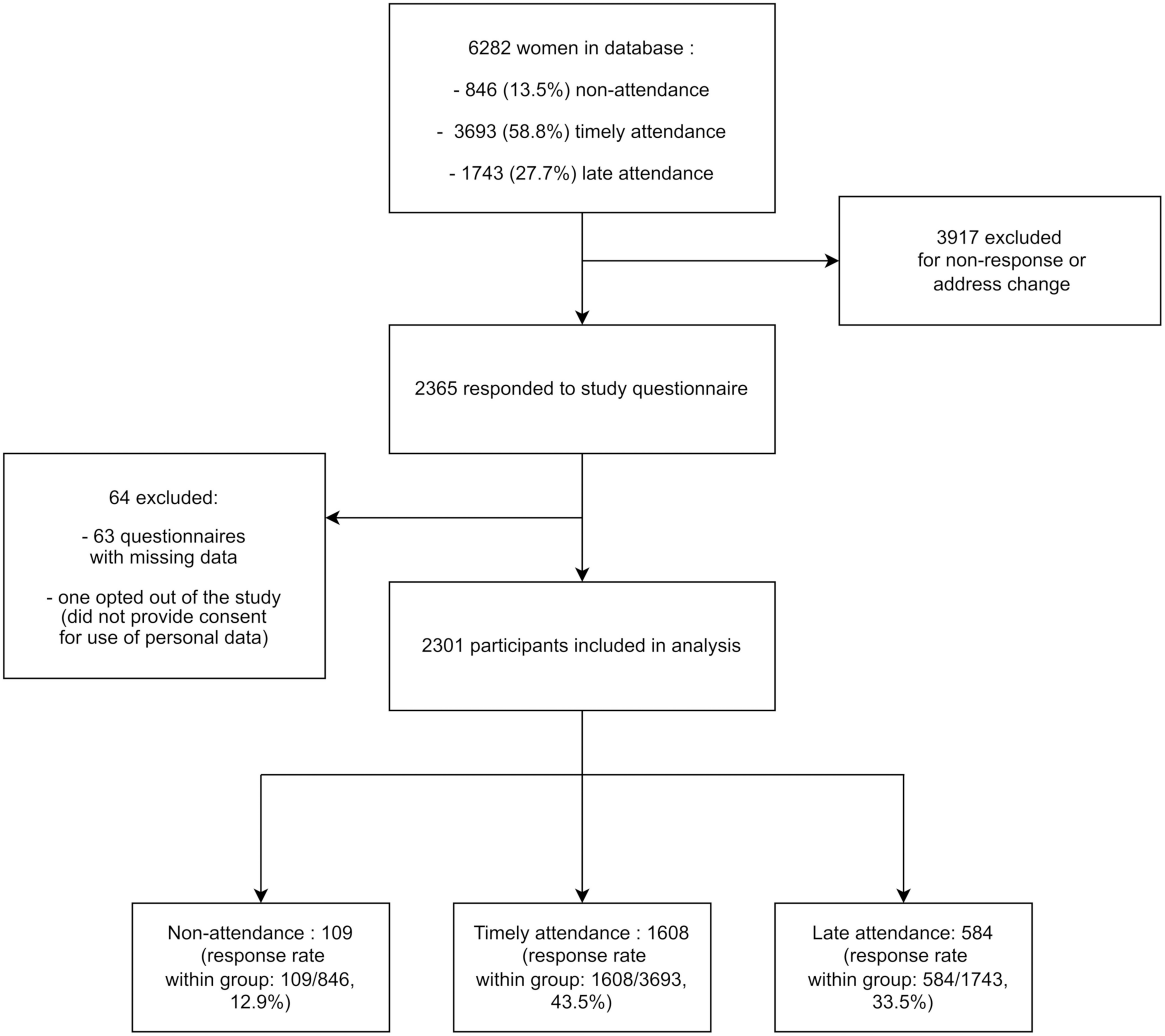
Study flowchart.

Overall, 6282 women were identified from the regional center for OBCS coordination database. Among these women, 846 (13.5%) were non‐attenders, 1743 (27.7%) were late attenders, and 3693 (58.8%) were timely attenders. A total of 2301 responders (overall response rate 36.6%) were included in the study; namely 109 women (response rate within group 109/846: 12.9%) who had not attended the organized mammography (participants with no attendance), 584 (response rate within group 584/1743: 33.5%) who had performed their mammography late (participants with late attendance), and 1608 women (response rate within group 1608/3693: 43.5%) who underwent mammography promptly (Figure 1). The mean age of participants was 62.8 ± 6.6 years. Most participants did not have a high school diploma or higher education status (60.1%). Baseline characteristics are detailed in Table 1. Based on univariate analysis, the factors associated with prompt attendance to OBCS were older age (*p* < 0.0001), absence of high school diploma (*p* = 0.004) and socio‐professional status (*p* = 0.0003) (Table 1). People who were unemployed or seeking employment or who never worked (4.9%) were underrepresented in the prompt attendance category. Administrative, sales, and service workers had a higher probability of belonging to the late attendance category (29.4%). Retired people constituted 51.2% of the prompt attendance category, which was higher than for the other categories. Moreover, having a declared GP who encouraged them to participate in OBCS (*p* = 0.008), having a regular gynecological follow‐up (every 5 years) (*p* < 0.0001) and prior participation in a colorectal cancer screening program (*p* < 0.0001) were also associated with prompt screening attendance (Table 2).

**TABLE 1:** Baseline characteristics of women invited for organized breast cancer screening that responded to the study questionnaire, according to the type of attendance (prompt attendance, delayed attendance, or non‐attendance).

	Participants that were invited to an organized breast cancer screening mammography, *n* (%)				*p*‐value
	No attendance *n* = 109	Late attendance *n* = 584	Prompt attendance *n* = 1608	Total *n* = 2301	
Age, years (mean ± SD)	62.1 ± 7.2	61.8 ± 6.7	63.3 ± 6.5	62.8 ± 6.6	<0.0001
Education status (high school diploma or higher)					
Yes	55 (50.5)	272 (46.6)	642 (39.9)	969 (42.1)	0.004
No	54 (49.5)	312 (53.4)	966 (60.1)	1332 (57.9)	
Marital status as of June 2020					
Married or living maritally	74 (67.9)	409 (70.0)	1137 (70.7)	1620 (70.4)	0.80
Single, separated, divorced, widowed	35 (32.1)	175 (30.0)	471 (29.3)	681 (29.6)	
Having children					
Yes	101 (92.7)	531 (90.9)	1441 (89.6)	2073 (90.1)	0.73
No	8 (7.3)	53 (9.1)	167 (10.4)	228 (9.9)	
Socio‐professional status since June 2020					
Farmers/agricultural workers	6 (5.5)	12 (2.0)	30 (1.9)	48 (2.1)	0.0003
Craft workers, retailers, and business owners	5 (4.6)	16 (2.7)	37 (2.3)	58 (2.5)	
Higher grade professional, administrative and managerial position	10 (9.2)	58 (9.9)	129 (8.0)	197 (8.6)	
Intermediate occupations	5 (4.6)	29 (5.0)	95 (5.9)	129 (5.6)	
Administrative/sales/service workers	22 (20.2)	172 (29.4)	353 (21.9)	547 (23.8)	
Manual laborer	2 (1.8)	14 (2.4)	61 (3.8)	77 (3.3)	
Retired	48 (44.0)	246 (42.1)	824 (51.2)	1118 (48.6)	
Unemployed/seeking employment/never worked	11 (10.1)	37 (6.3)	79 (4.9)	127 (5.5)	
Nationality					
French‐born	101 (92.7)	561 (96.1)	1537 (95.6)	2199 (95.6)	0.35
Naturalized French	5 (4.6)	14 (2.4)	53 (3.3)	72 (3.1)	
Nationality other than French	3 (2.7)	9 (1.5)	18 (1.1)	30 (1.3)	
Population density of residence^a^					
Low	33 (45.2)	169 (43.8)	471 (43.5)	673 (43.6)	0.96
Very low	40 (54.8)	217 (56.2)	612 (56.5)	869 (56.4)	
Potential score of local healthcare services accessibility median, median [quartiles Q1, Q3]	2.83 [1.71 to 3.45]	2.65 [1.63–3.45]	2.61 [1.63 to 3.45]	2.65 [1.63 to 3.45]	0.46
Index of social disadvantage, median [quartiles Q1, Q3]	0.62 [−0.05 to 1.29]	0.62 [−0.06 to 1.23]	0.36 [−0.06 to 1.18]	0.42 [−0.06 to 1.18]	0.11

^a^ Low corresponds to municipalities where less than 50% of the population lives outside an urban center; “very low” corresponds to municipalities where more than 50% of the population lives outside an urban center. Because the Aube department is sparsely populated, these two categories are sufficient to describe its density.

**TABLE 2:** Factors associated with the type of attendance (prompt attendance, delayed attendance, or non‐attendance) for women who were invited to an organized breast cancer screening.

	Participants that were invited to an organized breast cancer screening (OBCS) mammography, *n* (%)				*p*‐value
	No attendance *n* = 109	Late attendance *n* = 584	Prompt attendance *n* = 1608	Total *n* = 2301	
Have had a designated general practitioner (GP)					
Yes	105 (96.3)	567 (97.1)	1569 (97.6)	2241 (97.4)	0.64
No	4 (3.7)	17 (2.9)	39 (2.4)	60 (2.6)	
Frequency of GP consultations					
Once every 3 months, or more often	48 (44.0)	254 (43.5)	772 (48.0)	1074 (46.7)	0.15
Less than once every 3 months	61 (56.0)	330 (56.5)	836 (52.0)	1227 (53.3)	
GP encouragement for participant to attend OBCSs					
Yes	77 (70.6)	466 (79.8)	1322 (82.2)	1865 (81.0)	0.008
No	32 (29.4)	118 20.2)	286 (17.8)	436 (19.0)	
GP gender					
Female	52 (47.7)	241 (41.3)	659 (41.0)	952 (41.4)	0.38
Male	57 (52.3)	343 (58.7)	949 (59.0)	1349 (58.6)	
Had a gynecological follow‐up at least once every 5 years by a physician or midwife					
Yes	49 (44.9)	403 (69.0)	1180 (73.4)	1632 (70.9)	<0.0001
No	60 (55.1)	181 (31.0)	428 (26.6)	669 (29.1)	
Owning motorized transport (car, motorbike)					
Yes	96 (88.1)	542 (92.8)	1498 (93.2)	2136 (92.8)	0.24
No	13 (11.9)	42 (7.2)	109 (6.8)	164 (7.2)	
Travel time to nearest mammography provider					
Less than 30 min	79 (72.5)	440 (75.3)	1248 (77.6)	1767 (76.8)	0.30
More than 30 min	30 (27.5)	144 (24.7)	360 (22.4)	534 (23.2)	
Personal history of cancer other than breast cancer					
Yes	15 (13.8)	59 (10.1)	166 (10.3)	240 (10.4)	0.50
No	94 (86.2)	525 (89.9)	1442 (89.7)	2061 (89.6)	
Had close relatives or friends who had breast cancer in the past					
Yes	56 (51.4)	290 (50.0)	869 (54.0)	1215 (52.8)	0.18
No	53 (48.6)	294 (50.3)	739 (46.0)	1.086 (47.2)	
Participated at least once in an organized colorectal cancer screening					
Yes	68 (62.4)	452 (77.4)	1313 (81.6)	1833 (79.7)	<0.0001
No	41 (37.6)	132 (22.6)	295 (18.4)	468 (20.3)	

Abbreviations: GP, general practitioner; OBCS, organized breast cancer screening.

By multivariable analysis, older participants were significantly less likely to attend late (OR 0.96 [95% CI 0.94 to 0.98], *p* < 0.0001) or to refrain from screening (OR 0.93 [95% CI: 0.89 to 0.97], *p* = 0.0005). Women who did not have a regular gynecological follow‐up were more likely to attend late (OR 1.77 [95% CI 1.34 to 2.34], *p* < 0.0001) or not to undergo screening (OR 5.40 [95% CI: 3.17 to 9.19], *p* < 0.0001). Women who did not participate in colorectal cancer screening were more likely to screen later (OR 1.34 [95% CI: 1.00 to 1.79], *p* = 0.04) or to refrain from attending the recommended mammography (OR 2.46 [95% CI: 1.45 to 4.16], *p* = 0.0008) in comparison to women who promptly attended the OBCS mammography (Table 3). A higher education status (OR 1.30 [95% CI: 1.02 to 1.66], *p* = 0.03) and living in an area with a higher social disadvantage index (OR 1.14 [95% CI: 1.01 to 1.28], *p* = 0.03) were significantly associated with late attendance. There was significantly collinearity between GP consultation frequency and GP recommendation, so the GP recommendation was included due to its lower *p*‐value. Similarly, the information about socio‐professional status was already covered by the inclusion of education status and the index of social disadvantage.

**TABLE 3:** Multinomial and multivariable logistic regression analysis of the factors associated with late attendance or no attendance compared to prompt attendance at an organized breast cancer screening (OBCS) mammography.

	Participants that were invited to an organized breast cancer screening (OBCS) mammography			
	No attendance		Late attendance	
	OR [95% CI]	*p*‐value	OR [95% CI]	*p*‐value
Age	0.93 [0.89 to 0.97]	0.0005	0.96 [0.94 to 0.98]	<0.0001
Education status (high school diploma or higher)				
No	1.00 (ref)		1.00 (ref)	
Yes	1.21 [0.73 to 2.01]	0.45	1.30 [1.02 to 1.66]	0.03
GP encouragement to participate in the OBCS				
No	1.00 (ref)		1.00 (ref)	
Yes	0.80 [0.43 to 1.49]	0.48	1.04 [0.75 to 1.44]	0.81
Frequency of gynecological follow‐up				
At least once every 5 years	1.00 (ref)		1.00 (ref)	
Less than once every 5 years	5.40 [3.17 to 9.19]	<0.0001	1.77 [1.34 to 2.34]	<0.0001
Had close relatives or friends who had breast cancer in the past				
No	1.00 (ref)		1.00 (ref)	
Yes	0.89 [0.54 to 1.46]	0.64	0.88 [0.70 to 1.12]	0.59
Never participated in an organized colorectal cancer screening				
No	1.00 (ref)		1.00 (ref)	
Yes	2.46 [1.45 to 4.16]	0.0008	1.34 [1.00 to 1.79]	0.04
Index of social disadvantage	1.04 [0.81 to 1.32]	0.77	1.14 [1.01 to 1.28]	0.03

*Note*: The reference group was women who attended the OBCS mammogram promptly.

Abbreviations: GP, general practitioner; OBCS, organized breast cancer screening; OR, odds ratio.

The response rate of participants for the last question of the questionnaire was lower for those with late attendance (*n* = 71/584, 12.2%), when compared with the “no attendance” group (*n* = 69/109, 63.3%). Women who had not undergone mammography mostly cited a lack of interest in health matters (15.9%, *p* = 0.007) and lack of comprehension about the utility of undergoing mammography every 2 years (14.5%, *p* = 0.004) to be the main reasons for their lack of attendance (Table 4). In comparison, women with late attendance more frequently cited a lack of time (43.7%, *p* = 0.03) as the reason for their delay (Table 4).

**TABLE 4:** Reasons cited by participants for late or non‐attendance at an organized breast cancer screening (OBCS) mammography.

	Participants that were invited to an organized breast cancer screening (OBCS) mammography		
	No attendance, *n* = 69	Late attendance, *n* = 71	*p*‐value
Fear of getting COVID‐19 at the mammography providers site			
Yes	21/69 (30.4)	24/71 (33.8)	0.67
No	48/69 (69.6)	47/71 (66.2)	
Psychological repercussions of the COVID‐19 pandemic and first lockdown (anxiety, depression or burn out)			
Yes	12/69 (17.4)	11/71 (15.5)	0.76
No	57/69 (82.6)	60/71 (84.5)	
Job loss or reduced income during the COVID‐19 pandemic			
Yes	1/69 (1.4)	1/71 (1.4)	1.00
No	68/69 (98.6)	70/71 (98.6)	
Difficulty with transportation			
Yes	14/69 (20.3)	8/71 (11.3)	0.14
No	55/69 (79.7)	63/71 (88.7)	
Too far from the nearest mammography provider			
Yes	7/69 (10.1)	4/71 (5.6)	0.32
No	62/69 (89.9)	67/71 (94.4)	
Fear that a mammogram would detect breast cancer			
Yes	6/69 (8.7)	13/71 (18.3)	0.10
No	63/69 (91.3)	58/71 (81.7)	
Discomfort caused by mammogram			
Yes	25/69 (36.2)	19/71 (26.8)	0.23
No	44/69 (63.8)	52/71 (73.2)	
Lack of time			
Yes	18/69 (26.1)	31/71 (43.7)	0.03
No	51/69 (73.9)	40/71 (56.3)	
Not ascribing importance to health‐related issues			
Yes	11/69 (15.9)	2/71 (2.8)	0.007
No	58/69 (84.1)	69/71 (97.2)	
Did not understand the utility of undergoing mammogram every 2 years			
Yes	10/69 (14.5)	1/71 (1.4)	0.004
No	59/69 (85.5)	70/71 (98.6)	
Having heard controversies surrounding organized breast cancer screening			
Yes	4/69 (5.8)	4/71 (5.6)	1.00
No	65/69 (94.2)	67/71 (94.4)	

## 4 DISCUSSION

Our results identify numerous participant characteristics that were significantly associated with deferral or non‐performance of OBCS during the COVID‐19 pandemic in the Aube Department of France. The main factor identified was the absence of regular gynecological follow‐up, which was associated with an increased risk of deferral, and an even higher increase in the risk of non‐performance. Similarly, the absence of any history of prior participation in colorectal cancer screening was also associated with a risk of delayed performance, and more strongly with the risk of non‐attendance. Younger age was associated with both delayed performance and non‐performance, while living in a socially disadvantaged area as well as a higher educational status were both associated with deferral.

Some of our findings are in line with the literature. Factors such as gynecological follow‐up,^31^, ^32^, ^33^ participation in colorectal cancer screening,^34^, ^35^, ^36^, ^37^ social deprivation and rural residence^32^, ^38^, ^39^ as well as socio‐economic status^32^, ^40^, ^41^ have all been commonly reported as being related to lower participation in OBCSs. Various studies have also identified other factors such as age, marital status or number of children.^39^, ^40^, ^41^, ^42^, ^43^ However, past systematic reviews have pointed out the difficulty of summarizing the contextual factors affecting OBCS uptake due to the heterogeneity of contexts, places, periods and methodologies across studies.^32^, ^40^

Previous studies such as Rollet et al. (2021) and Plourde et al. (2016), have shown that women who consult a physician (from any medical specialty) are more likely to participate in OBCS, especially if the physician recommends the screening and is a skilled communicator.^31^, ^32^, ^42^ Moreover, the consulting physician's specialty affects the strength of the association between medical follow‐up and screening uptake with gynecologists having a predominant role.^31^, ^33^, ^42^ It has also been reported that participation in one form of cancer screening (breast or cervical cancer) is strongly correlated with participation in other screening types.^35^

In France, gynecologists perform most screening exams for cervical cancer, and parameters such as the frequency of gynecological follow‐up and the number of gynecologists per capita have been found to be related to improved participation in cervical cancer screening.^44^ A cohort study reported that a greater density of gynecologists within a 5 km radius of the GP's office was associated with a higher rate of participation in cervical cancer screening.^45^ Although the reason for this is not known, it seems reasonable to assume that gynecologists are able to deliver relevant and up to date information to encourage screening. The frequency of gynecological follow‐up may be interpreted as a proxy for adherence to care, and also as a surrogate indicator of interest in cancer screening (for cervical cancer, and for breast cancer).

Previous literature was also consistent with our findings regarding the prior participation of women in organized colorectal cancer screening.^34^, ^35^, ^36^, ^37^ In our questionnaire, prior participation was presented as a binary question which may have reinforced the strength of the association between colorectal and breast cancer screening. Two studies from France and England have shown that participation in all three forms of screening (breast, colorectal and cervical cancer) are significantly related.^34^, ^46^ Therefore, promoting one type of screening might automatically enhance participation in the others.

There are conflicting findings on the impact of age on screening uptake. Some studies have shown that older age is associated with lower participation,^42^, ^47^, ^48^, ^49^ while more recent studies show a positive effect of age.^50^, ^51^, ^52^, ^53^ The most recent systematic reviews have failed to find evidence of any association between screening uptake and age.^32^, ^40^ The youngest women eligible for OBCS are often professionally active. The employment rate among women for OBCS increased substantially between 1975 and 2018.^54^ It is therefore possible that the reasoning of a lack of time is plausible for non‐ or late attendance among women who are still working or too young to have retired, as previously reported in another French study.^53^ It may also be worth noting that the mean age of women who performed their mammography promptly was 1 year older than the official retirement age in France in 2022.^55^ A French study reported that when retired women participate in organized or individual breast cancer screening, they do so more regularly than working women.^56^ As Shneyderman et al. (2016) suggested, with an almost universal access to the internet and the widespread use of new technologies such as smartphones, older women eligible for OBCS may have been better informed than women of comparable ages in the 2000s.^57^ However, although a study has shown that internet use was associated with cancer‐preventive behaviors in older adults, there was no association with breast cancer screening.^58^

We found that women with a higher educational status were more likely to delay their OBCS mammography. Previous studies have reported that women with 11 to 15 years of schooling are more likely to participate in screening than those with lower (10 years or fewer) or higher (>15 years) educational status.^47^, ^59^ It has also been postulated that women with lower educational status have greater difficulty obtaining and understanding health‐related information.^47^, ^59^ Conversely, highly educated women (e.g., post‐doctorate) are busier, better able to assess the risk/benefit ratio of OBCS, and more often have an individual screening than others.^60^ A study conducted in urban settings in southern France has shown that managerial status is negatively correlated with organized screening.^61^ One meta‐analysis found that having an intermediate education status (neither very low nor very high) was associated with greater OBCS uptake compared to a lower level, but there was no significant differences with a higher education status.^40^ Although familial or genetic determinants were not specifically explored in this study, having social ties with people who had cancer did not appear to be a major predictor of screening.

Lastly, we also showed that women who live in socially disadvantaged areas are at high risk of delaying OBCS, which is again concordant with previous studies.^38^, ^50^, ^62^ In contrast with previous studies, we did not find any association between attendance and the distance from the participant's home to the nearest mammography provider.^32^, ^38^ These elements point to the possibility that the social context of the area of residence could present a greater obstacle than the geographical access to care facilities. Innovative studies including those by Trivedi et al. (2022) and Guillaume et al. (2017) have reported that mobile mammography screening is a useful means to mitigate the influence of rural living and social deprivation on OBCS uptake.^63^, ^64^ The findings in our article may not apply in low‐ and middle‐income countries, as access to care and program characteristics tend to play a bigger role in these contexts.^65^

Fear of contracting COVID‐19 at the mammography provider's site was one of the most common reasons indicated both for not undergoing OBCS (21/69, 30.4%) and for a delayed OBCS (24/71, 33.8%) in our study. Discomfort was also a major barrier, cited as a reason for not attending in 36.2% of non‐attenders but was not a significant result in our study. Preventing this discomfort could require technological developments but also empathy and reassurance during the examination. This is in line with the inconsistent findings in the literature on the link between discomfort and participation in screening.^66^, ^67^

Regarding the limitations of our study, the response rate was average, with 36.6% of all eligible women returning a valid questionnaire. There was potential for selection bias in our findings, since women had to have participated in the prior round of screening in 2018 to 2019 and to have been invited in the second half of 2020 to be eligible. Therefore, women who never participated or who had not participated in a long time were not included in this study. Further studies could ascertain whether the factors associated with attendance in this population are the same as for women who have already undergone screening.^68^ Second, there was potential for recall bias in that the information was self‐reported by patients in retrospect.^69^ We cannot exclude the possible existence of other confounding factors that were not measured in our study, although the use of a multivariable model allowed us to control for potential confounding.

Only a minority of eligible women answered the last question of the questionnaire, which pertained to the reasons for delaying or failing to attend screenings. We hypothesize that many of the participants who performed their screening late were not aware that they were considered as “late attenders” and therefore did not feel concerned by this question, perhaps believing that they had attended in a timely manner.

## 5 CONCLUSION

Our study identified a number of factors related to the participation in OBCS, which may ultimately be leveraged to enhance uptake rates. Compared to women who promptly underwent mammography, non‐ and late‐screening participants were younger, had less frequent gynecological follow‐up and a less frequent history of colorectal cancer screening, while women with higher education status and those residing in socially disadvantaged areas were more likely to attend late. Discomfort was often cited as a reason for delaying OBCS or not undergoing screening. Therefore, interventions aiming to improve screening uptake may need to address three main issues: finding the optimal frequency of reminders in order to encourage women to undergo OBCS (these reminders could be delivered by healthcare professionals); improving the healthcare system's ability to deliver timely specialized information to women that reach the age for screening (the information must be relevant and include the latest evidence, in line with the type of information delivered by gynecologists); and avoiding discomfort for women undergoing screening (with empathy, reassurance and technological development of mammography solutions that limit discomfort).

## AUTHOR CONTRIBUTIONS

**Jean Papadacci Stephanopoli:** Conceptualization (lead); data curation (lead); investigation (lead); methodology (lead); supervision (lead); validation (lead); writing – original draft (lead); writing – review and editing (lead). **Leila Bouazzi:** Data curation (equal); formal analysis (equal); writing – original draft (equal). **Myriam Guerbaz‐Sommi:** Resources (lead); supervision (lead); writing – review and editing (lead). **Olivier Graesslin:** Writing – review and editing (lead). **Aline Hurtaud:** Writing – review and editing (equal). **Salvatore Ilardo:** Conceptualization (equal); methodology (equal); supervision (equal). **Jan Chrusciel:** Writing – review and editing (equal). **Cecile Etude Barbaret:** Conceptualization (equal); methodology (equal); resources (equal); supervision (equal). **Camille Bertrand:** Conceptualization (equal); methodology (equal); resources (equal); supervision (equal). **Stéphane Sanchez:** Conceptualization (lead); investigation (lead); methodology (lead); supervision (lead); writing – original draft (lead); writing – review and editing (lead).

## FUNDING INFORMATION

No funding to declare.

## CONFLICT OF INTEREST STATEMENT

The authors have no relevant financial or non‐financial interests to disclose.

## CONSENT

Informed consent was obtained from all individual participants included in the study.

## Supporting information


Table S1.

## Data Availability

Data are available upon request by the corresponding author.
