# Supplementary material for: Factors associated with deferral or non‐performance of an organized breast cancer screening program during the COVID‐19 pandemic in France
Source: Cancer Med. 2024 Aug 27;13(16):e7444. doi: 10.1002/cam4.7444 (PMC11349821; doi:10.1002/cam4.7444)
Supplement: Supplementary file 1 — Table S1. [file CAM4-13-e7444-s001.docx]

**Supplementary Information**

**Supplementary Table 1.** Study Questionnaire

| **Question Number** | **Response options** | **Question type** |
| --- | --- | --- |
| 1. Do you have a declared general practitioner (GP)? | Yes/No | Binary |
| 2. On average, how often do you see your GP? | Several times a month/Between once a month and once every 3 months/Between once every 4 months and once every 12 months/Less than once a year | Multiple choice |
| 3. Does your GP encourage you to attend the mammogram proposed in the breast cancer screening program? | Yes/No | Binary |
| 4. Is your GP a man or a woman? | Male/Female | Binary |
| 5. Do you have gynecological follow-up at least once every 5 years, with a physician (GP or gynecologist) or midwife? | Yes/No | Binary |
| 6. Please indicate the highest level of educational qualification you have attained. | No diploma, primary school certificate/Middle school certificate/High school diploma/post-high-school diploma (high school +2 years)/Bachelor’s degree (3-year degree)/Masters 1 degree (4-year degree)/Masters 2 degree or engineering degree (5-year degree)/Post doctorate | Multiple choice |
| 7. Please indicate your marital status during the second semester of 2020 (at the time when you received the invitation to attend organized breast cancer screening). | Married or civil union/Living maritally/Divorced or separated/Widowed/Single | Multiple choice |
| 8. Do you have children? | Yes/No | Binary |
| 9. What was your socio-professional category during the second semester of 2020? | Farmer or agricultural workers/craft workers, retailers, or business owners/higher grade professional, administrative & managerial occupations/intermediate professions/administrative/sales/service workers/manual laborer/retired/unemployed, seeking employment or never worked | Multiple choice |
| 10. What is your nationality? | French-born nationality/Naturalized French /Other nationality | Multiple choice |
| 11. Do you have your own motorized transport (car, truck, motorbike)? | Yes/No | Binary |
| 12. How long does it take to travel to the nearest mammography provider from where you live? | Less than 15 minutes/Between 15 and 30 minutes/Between 30 minutes and 1 hour/More than 1 hour | Multiple choice |
| 13. Do you currently have, or have you ever had cancer other than breast cancer? | Yes/No | Binary |
| 14. Does anyone among your close relatives and friends have (or have they ever had) breast cancer? | Yes/No | Binary |
| **The following question is only for women who have not yet attended the screening mammogram after receiving the invitation, and for those who attended more than 26 months after the previous mammogram** | | |
| 15. Please indicate the reason(s) why you have not yet attended, or why you attended the mammography late.  Note: you may tick more than one reason. | Fear of contracting COVID-19 at the location of the mammography provider, Psychological repercussions (anxiety, depression or burnout) from the pandemic and first lockdown, Lost job or income during the pandemic, Difficulties travelling, Distance from home to nearest radiology office, Fear that mammogram might detect breast cancer, Discomfort caused by mammogram, Lack of time, Don’t accord much importance to health matters, Don’t understand the utility of doing a mammogram every 2 years, You've heard the controversies surrounding organized breast cancer screening. | Multiple choice |
